# Double-Vestibular Incision Tunnel Technique With Platelet-Rich Fibrin and Connective Tissue Graft for Mandibular Anterior Gingival Recession: A Case Series

**DOI:** 10.7759/cureus.105985

**Published:** 2026-03-27

**Authors:** Balakumaran Maroudaviran, Mohanagowri Narayanan, Kennedy Babu, Devanand Gandhimadhi, Arunagiri Karunanithi

**Affiliations:** 1 Periodontics, Mahatma Gandhi Postgraduate Institute of Dental Sciences, Pondicherry, IND

**Keywords:** connective tissue graft, double-vestibular incision subperiosteal tunnel access, gingival recession, platelet-rich fibrin, root coverage

## Abstract

Gingival recession (GR), also known as the migration of the gingival margin in an apical direction beyond the cementoenamel junction (CEJ), is not merely an esthetic problem. It may also cause cervical lesions, increased susceptibility to root-surface caries, hypersensitivity, and compromised periodontal health. These defects can be multifactorial and often occur more commonly in the mandibular anterior region. Due to the technique sensitivity of standard approaches, there is a growing inclination towards minimally invasive techniques for managing multiple GR defects, especially in this area. Two systematically healthy male patients, aged 39 and 45 years, reported with multiple recession-type (RT) 1 and RT2 gingival recessions in the mandibular anterior region. Both cases underwent the novel double-vestibular incision subperiosteal tunnel access (double-VISTA) technique with placement of platelet-rich fibrin (PRF) and subepithelial connective tissue graft (SCTG), respectively. Double-VISTA, along with PRF or SCTG, proved to be a reliable procedure for the management of multiple GR defects in the mandibular anterior region. Both cases showed excellent improvements in clinical parameters. Within the limitations of the case series, PRF may serve as an effective substitute for SCTG in the double-VISTA approach. However, further larger-sample-size studies are required to confirm its sustainability and predictability.

## Introduction

An esthetic smile is a reflection of both the oral and general health of a person [1]. Any imbalance between the gingival and the dental components can lead to an unesthetic appearance. With increasing awareness and demand for cosmetic enhancement, the management of the pink esthetic component has become a key concern in contemporary periodontal and esthetic dentistry [2].

Gingival recession (GR), by definition, is the migration of the gingival margin in a downward (apical) direction beyond the cementoenamel junction (CEJ), which causes root-surface exposure. This condition not only poses an esthetic challenge but also introduces functional complications, such as hypersensitivity due to root exposure, root-surface caries, cervical lesions, and the risk of further attachment loss [3].

GR is multifactorial; while inflammatory periodontal disease remains the primary cause, other contributing factors include traumatic toothbrushing, thin gingival phenotype, aberrant tooth positioning, high frenum attachment, and iatrogenic injuries [4]. Holistic management involves identifying and controlling these etiological factors before surgical correction. Root-coverage (RC) procedures are indicated only when periodontal health is stable, and there is an esthetic or functional need.

Over the years, different procedures have been suggested to obtain a predictable RC and harmonious tissue contours. Among these, the coronally advanced flap (CAF), often combined with a subepithelial connective tissue graft (SCTG), is considered the benchmark technique because of its well‑documented long‑term predictability [5]. However, CAF requires an adequate width of attached gingiva and incorporates vertical releasing incisions, which may affect healing and esthetic outcomes [6]. To overcome these limitations, tunnel and modified tunnel techniques were introduced, in which a split‑ or full‑thickness tunnel is prepared without vertical releasing incisions to allow placement of SCTG and coronal advancement of the soft tissues through a minimally invasive approach [7].

Tunnel techniques evolved from early pouch-and-tunnel approaches that enabled root coverage without vertical releasing incisions. Modified tunnel methods further refined flap mobilization and graft placement while preserving papillary integrity. The vestibular incision subperiosteal tunnel access (VISTA) technique represents an advanced iteration of these minimally invasive protocols. Zadeh introduced the VISTA technique [7]. In this technique, subperiosteal tunneling is done from a single-vestibular incision, which enables coronal advancement of the gingival margin without vertical releasing incisions, thereby preserving papillary integrity, minimizing scarring, and achieving superior esthetic results [8]. VISTA has shown favorable outcomes in terms of RC, increased gingival thickness, and improved width of keratinized tissue in the esthetic zone. The double-VISTA technique by Lin modifies VISTA to facilitate instrument access and graft placement in segments with multiple or quadrant‑wide recessions by using two vestibular access incisions at either end of the treated area [9].

The efficacy of tunnel-based techniques like double-VISTA largely depends on the choice of graft material placed within the subperiosteal tunnel. SCTG remains the gold standard graft material for RC but is associated with a second surgical site, increased operative time, and donor‑site morbidity [10]. Platelet-rich fibrin (PRF) eliminates donor-site morbidity, shortens operative time, and promotes early wound healing through sustained growth factor release, though its faster resorption may limit durability compared with SCTG [11]. However, comparative clinical data on the performance of PRF versus SCTG specifically within the double‑VISTA approach for mandibular anterior multiple GR are limited.

The present case series describes the clinical application of the double-VISTA technique combined with PRF and SCTG for treating multiple RT1 and RT2 GR in the mandibular anterior region. This approach focused on achieving a predictable root coverage, minimal surgical morbidity, and optimal esthetic outcomes through a minimally invasive protocol.

## Case presentation

Case 1 - double-VISTA with PRF

Patient Selection and Diagnosis

**Figure 1:**
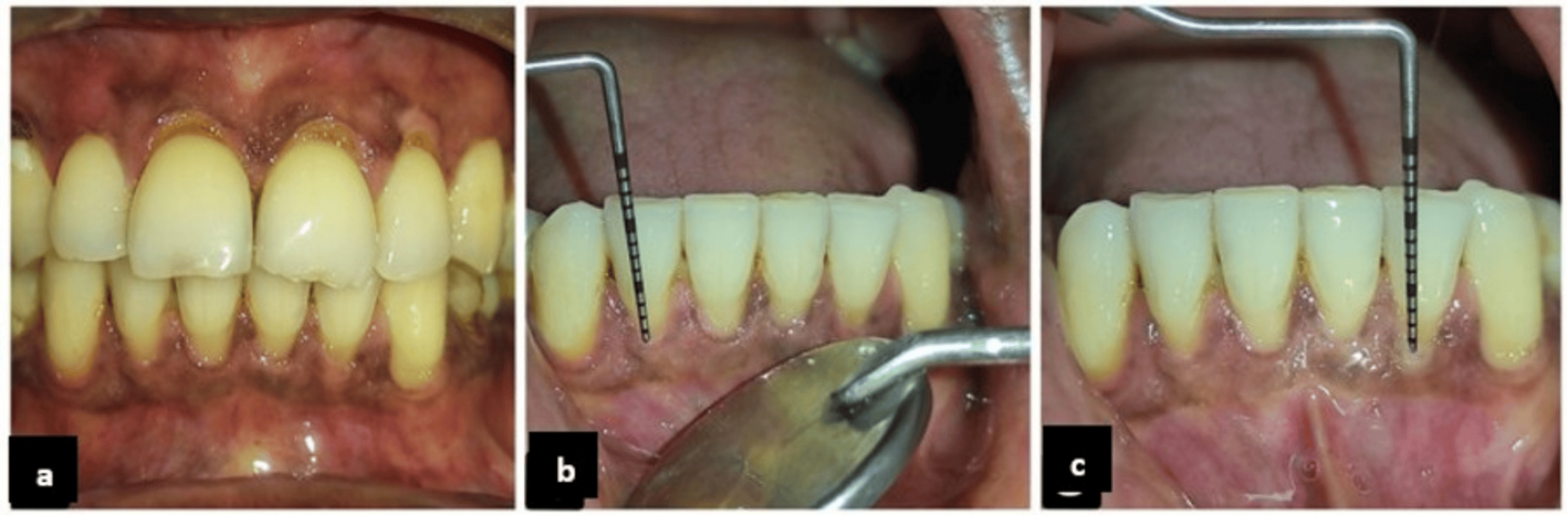
Preoperative clinical presentation and recession assessment (Case 1). (a) Preoperative image of (Case 1), (b) preoperative recession depth with respect to tooth 42, and (c) preoperative recession depth with respect to tooth 32.

A 45-year-old, systemically healthy male patient presented with the complaint of loss of gums in the mandibular front region. The patient had a history of aggressive horizontal toothbrushing twice daily. The patient had a history of mild sensitivity while drinking something cold, which resolved by itself. Clinical examination revealed multiple Cairo RT1 gingival recessions involving teeth #43, #42, #41, #31, #32, and #33, with measured recession depths (RD) of 1 mm in #43, 2.5 mm in #42, 3.5 mm in #41, and 2 mm in relation to #31, #32, and #33. Clinically, the probing depth of all teeth was 1 mm; the width of the attached gingiva was 0.5 mm with respect to teeth 43 and 33, 1.5 mm with respect to teeth 31 and 41, and 2 mm with respect to teeth 32 and 42. Also, the gingival phenotype was thin with respect to teeth 43 and 33 and thick with respect to teeth 31, 32, 42, and 41 (Figures 1a-1c).

Preoperative Phase

Initially, phase I therapy and oral hygiene reinforcement were done. Proper brushing technique was demonstrated. The patient was re-evaluated after one week, and informed consent for mucogingival surgery was obtained.

Surgical Procedure

**Figure 2:**
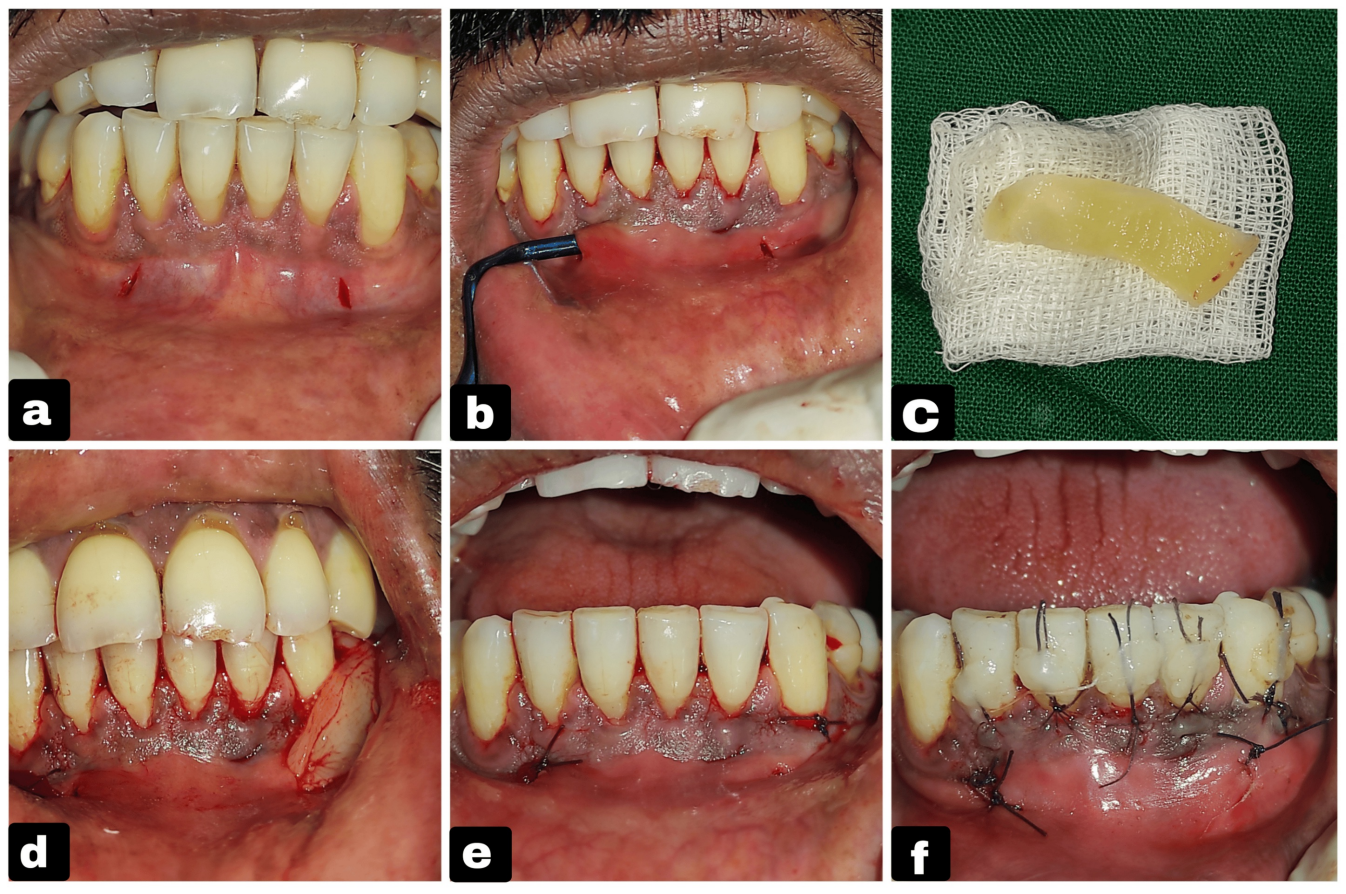
Surgical steps of the minimally invasive tunneling technique with PRF placement. (a) Double vestibular incision given. (b) Tunneling was done with the Vista instrument. (c) PRF was procured from the patient's blood. (d) PRF was placed inside the tunnel. (e) PRF membrane secured with sutures. (f) Coronal advancement of flap done using 6-0 Vicryl sutures and composite tags. PRF: platelet-rich fibrin

Following administration of local anesthesia (2% lidocaine with 1:80,000 epinephrine), two distinct vestibular access incisions were made - one distal to tooth #42 and the other distal to tooth #32. Both incisions were oriented vertically and measured approximately 2-3 mm in length to provide adequate access for instrumentation. At least 3 mm distance was maintained between the incision and marginal gingiva to minimize the risk of soft-tissue tearing during tunneling. A continuous tunnel was created subperiosteally between the two vestibular incisions using a VISTA-1 instrument with gentle lateral sweeping motions, then the tunnel was extended coronally toward the gingival margin with VISTA-2, VISTA-3, and VISTA-4 instruments, continuing one tooth beyond the recession sites to enable sufficient flap mobility and coronal repositioning. For this, the VISTA instrument from the GDC brand was used, which has a set of six instruments (VISTA 1, 2, 3, 4, 5, 6). VISTA-1 has a straight shank, which helps in the initial tunneling of the vestibular incision, whereas the other instruments have an angulated shank that helps in extending the tunnel into the sulcus region. Interproximal extension of the tunnel was performed beneath each papilla, avoiding any external papillary incisions to preserve vascularity and papillary integrity. A 10 mL sample of autologous blood was drawn from the patient in the middle of the procedure. Once the tunnel preparation is complete, the collected blood is transferred to a centrifuge (Remi C-854 machine; Mumbai, India: Remi Elektrotechnik Limited) within a minute to prevent growth factor loss, and once the PRF is formed, it is directly placed within the prepared tunnel and sutured to the flap. It was centrifuged at a speed of 1,300 rotations per min for 10 min to obtain PRF, which was then placed within the tunnel. The PRF was pierced on one end with a suture needle and guided into the tunnel by passing the suture needle under the tunnel through the vestibular incision, and then the end was sutured to the flap; similarly, the other end of the PRF was also sutured to the flap. Hence, both ends are secured to the flap. The tunnel was repositioned in the coronal position and stabilized with 6-0 Vicryl sutures and composite resin tags. Simple interrupted sutures were given to close vestibular incisions, and a periodontal dressing was placed. Periodontal dressing was used in this case as a protection from external contamination and to avoid the chance of debonding of composite resin, and the dressing was a non-eugenol co-pack (Figures 2a-2f).

Postoperative Management

Postoperatively, the patient was prescribed antibiotics (amoxicillin 500 mg TDS) and analgesics (paracetamol 500 mg TDS) for three days. An antibiotic was given as a prophylactic measure to reduce the risk of graft contamination. The patient was advised not to brush on the surgical site for two weeks. An antiseptic mouth rinse containing chlorhexidine (0.2%) was advised twice daily for two weeks. The periodontal pack was removed after one week, without disturbing the composite resin or the suture anchored to it. After two weeks, suture removal was performed, and the patient was reviewed at intervals of six months and one year for the assessment of the clinical parameters.

Clinical Outcomes

**Figure 3:**
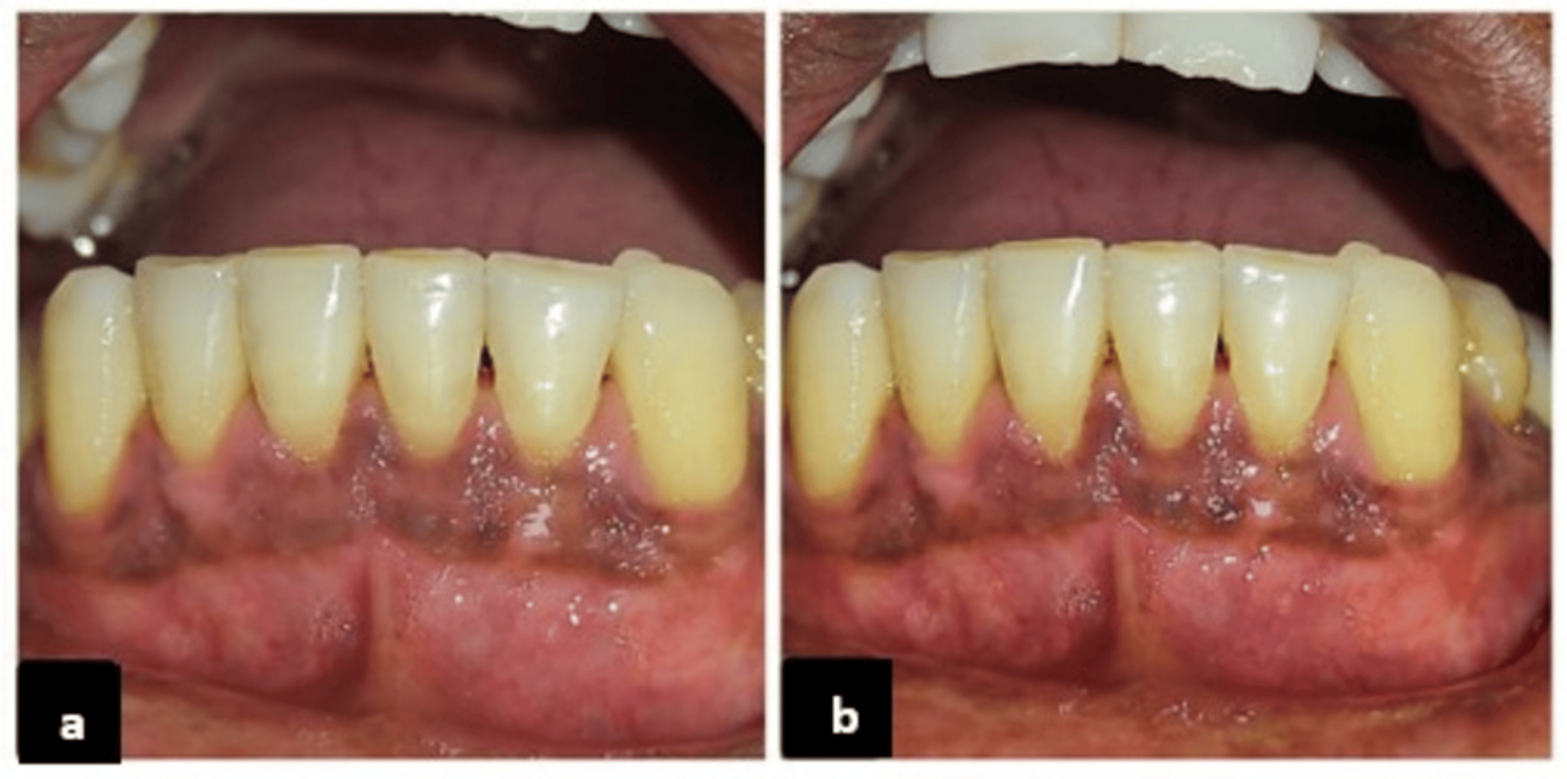
Long-term clinical evaluation of gingival healing after treatment. Gingival status at (a) six-month follow-up and (b) 12-month follow-up.

At six months, the RD was reduced to 0 mm for teeth #43, #42, #32, and #33, and to 0.5 mm for teeth #31 and #41. This corresponded to 100% root coverage in #43, #42, #32, and #33; 85% in #41; and 75% in #31, with a mean root coverage (RC) of 93.3%. At one year, a slight recurrence was observed, with RD increasing by 0.5 mm at #42 and #32, 2 mm at #41, and 1 mm at #31. The RC percentage reduced to 80% for #42, 75% for #32, and 50% for #41 and #31, resulting in a mean RC of 75.8%. Near complete RC was achieved on all four teeth. The patient reported complete resolution of tooth sensitivity. Esthetic satisfaction was reported as excellent (Figures 3a, 3b).

Case 2 - double-VISTA with subepithelial connective tissue graft

Patient Selection and Diagnosis

**Figure 4:**
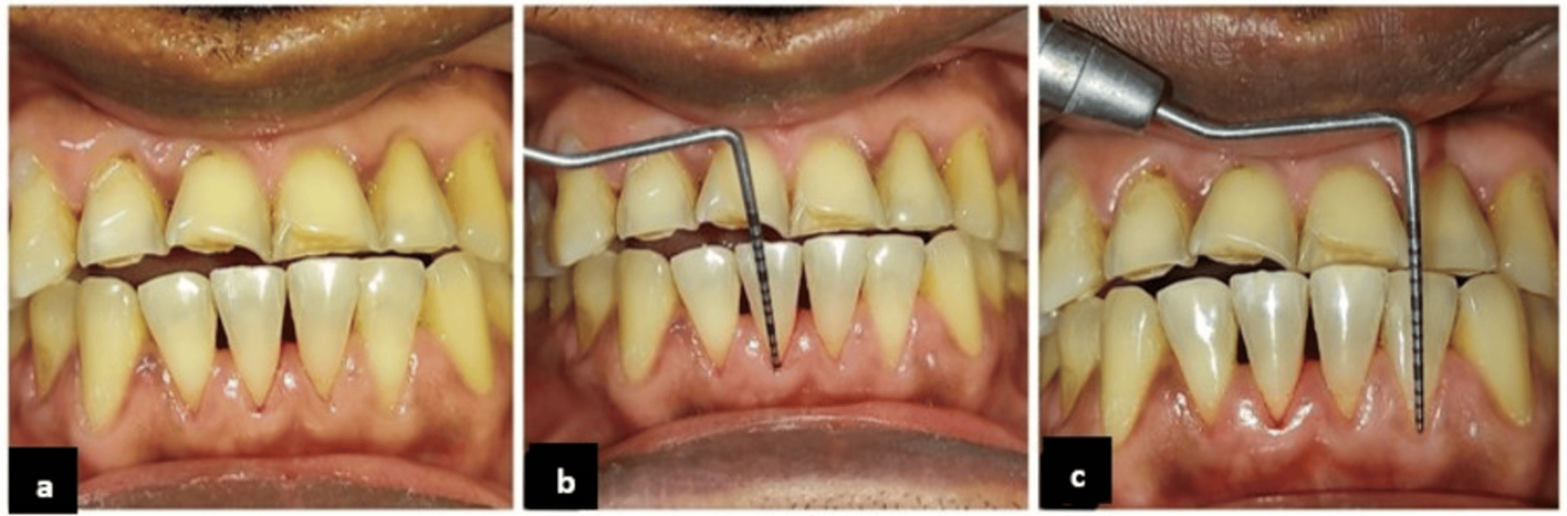
Preoperative clinical presentation and recession assessment (Case 2). (a) Preoperative image of (Case 2), (b) preoperative recession depth of tooth #41, and (c) preoperative recession depth of tooth #32.

A 39-year-old male, systemically healthy without a significant medical or surgical history, presented with multiple non-carious cervical lesions. Patient had a history of mild sensitivity occasionally on the site with no specific triggering or relieving factor association. It relieved itself, and the sensitivity was progressively increasing in nature. Clinical examination revealed Cairo RT2 GR involving teeth #42, #41, #31, and #32, each with a recession depth of 2 mm. The probing depth of all teeth was 1 mm, and the width of the attached gingiva was 2 mm for all teeth. The gingival phenotype was thick. The patient reported sensitivity and esthetic concerns (Figures 4a-4c).

Preoperative Phase

Initially, phase I therapy and oral hygiene reinforcement were done. Proper brushing technique was demonstrated. The patient was re-evaluated after one week, and informed consent for mucogingival surgery was obtained.

Surgical Procedure

**Figure 5:**
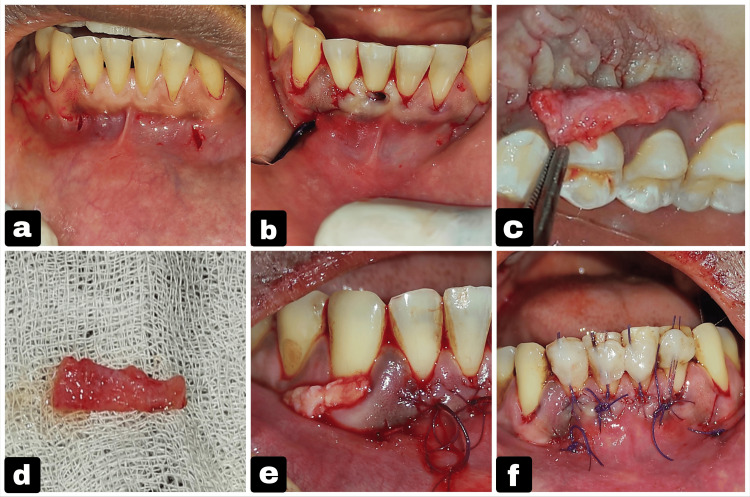
Surgical steps of the tunneling technique with SCTG for root coverage. (a) Double-vestibular incision given, (b) vestibular tunneling done, (c) SCTG harvested by trapdoor technique from palate, (d) harvested connective tissue, (e) SCTG placed inside the tunnel, and (f) coronal advancement of flap done for closure using 6-0 Vicryl sutures. SCTG: subepithelial connective tissue graft

The double-VISTA technique was performed as outlined earlier. SCTG was procured from the palate by the trapdoor technique and positioned within the prepared tunnel. The SCTG was pierced on one end with a suture needle and guided into the tunnel by passing the suture needle under the tunnel through the vestibular incision, and then the end was sutured to the flap; similarly, the other end of the SCTG was also sutured to the flap. Hence, both ends are secured to the flap. Repositioning of the flap was done coronally and secured with 6-0 Vicryl sutures and composite resin tags, while vestibular incisions were closed with simple interrupted sutures. A periodontal dressing was placed to protect the surgical site. Postoperative medications and hygiene instructions were provided. After two weeks, suture removal was done, and follow-up evaluations were done at six months and one year (Figures 5a-5f).

Postoperative Management

The postoperative management was similar to the previous case with a course of antibiotics and analgesics. Clinical evaluation was done at six months and one year.

Clinical Outcomes

**Figure 6:**
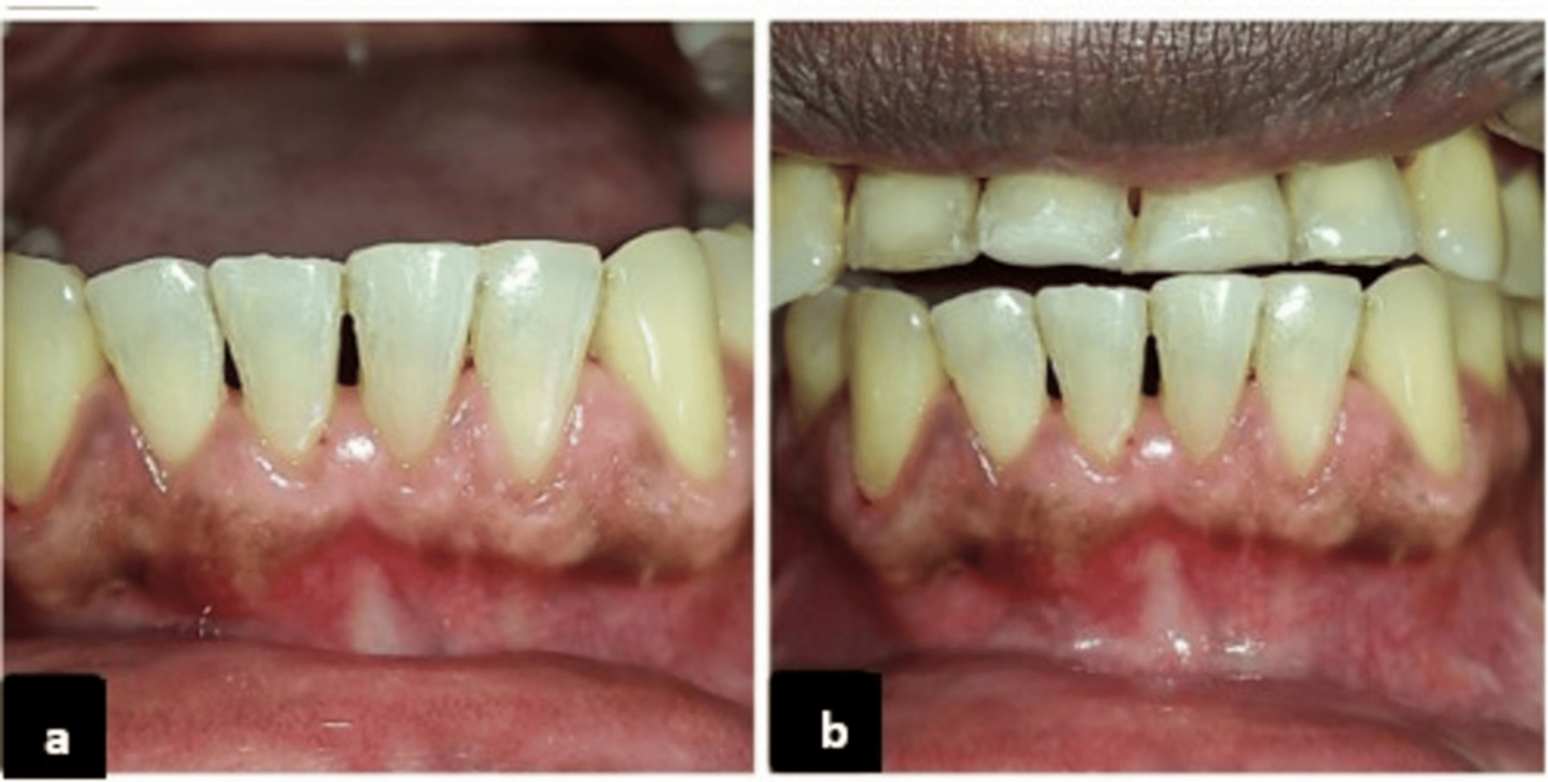
Postoperative gingival outcomes at follow-up intervals (Case 2). Gingival status at (a) six-month follow-up and (b) 12-month follow-up.

At six months, the RD was reduced to 0 mm for teeth #41, #42, and #31 and to 0.5 mm for #32, corresponding to 100% RC in teeth #41, #42, and #31 and 75% in #32, with a mean root coverage (MRC) of 93.7%. The results remained stable at the one-year follow-up, demonstrating satisfactory soft tissue healing and esthetic enhancement. Excellent color blending and natural contour noted. Tooth sensitivity has completely resolved. Esthetic satisfaction was excellent. The palatal donor site healed with minimal scarring (Figures 6a, 6b).

## Discussion

The outcome of any RC technique depends on several factors, including local anatomy, periodontal health, tissue phenotype, tension, design of flap, and the operator’s surgical skill. Among the various techniques for RC, the coronally advanced flap (CAF) combined with SCTG is traditionally considered the ideal choice due to its well-documented predictability. However, CAF requires adequate keratinized tissue and involves vertical releasing incisions, which may compromise esthetics and increase morbidity. Therefore, less invasive alternatives, such as the VISTA technique, gained attraction with their ability to achieve predictable root coverage with reduced trauma.

The VISTA approach offers several advantages through its remote vestibular incision, which minimizes disruption to the marginal gingiva and adjacent papillae. Subperiosteal tunnelling enables tension-free advancement of the flap coronally while preserving the papillary integrity, thus enhancing vascularity and minimizing postoperative micromotion. This technique has demonstrated favorable outcomes in terms of increased thickness of gingiva and width of keratinized tissue and esthetic integration [10].

Allen critically reviewed prevailing misconceptions about the usage of the tunnelling approach in the anterior region of the mandible and demonstrated that, with proper technique and graft adaptation, anatomical challenges such as a shallow vestibule or prominent frenum do not preclude successful outcomes [12]. Azaripour et al. conducted a double-blind randomized controlled trial (RCT) comparing CAF and the tunnel approach in combination with SCTG. They reported comparable RC and esthetic scores between the methods, with all patients expressing satisfaction and willingness to undergo similar procedures in the future [13]. Mansouri et al. also conducted a randomized trial comparing VISTA and CAF combined with SCTG and found that both approaches were equally effective in reducing gingival recession and improving clinical parameters [14]. While no significant differences were observed in mean RC, the VISTA group exhibited slightly higher complete root coverage and greater patient comfort [14]. Similarly, Fernández-Jiménez et al. compared modified VISTA (m-VISTA) with CAF for RT2 recessions and found an absence of statistically significant variation in RC percentages at six-month and one-year follow-ups. Nevertheless, m-VISTA showed superior flap stability and esthetic integration, underscoring its clinical advantages [15].

The VISTA technique also has some limitations when applied to extensive or full-quadrant recession defects. A single vestibular access incision may restrict instrument reach and complicate graft placement in distant areas. Lin proposed the double-VISTA modification to overcome this limitation, involving two separate vestibular incisions - one mesial to the most anterior tooth and another distal to the most posterior tooth within the treated segment. This dual-access design facilitates comprehensive subperiosteal tunnelling in both directions, allowing easier graft placement and enhanced adaptability for multiple or quadrant-wide recessions. Lin presented a case report utilizing this novel double-VISTA modification to manage multiple GR defects linked with cervical lesions of non-carious origin [16]. Their two-year follow-up demonstrated stable and complete RC, improved phenotype of the gingiva, and harmonious architecture of soft tissue, validating the consistency and durability of the technique [16].

Both cases showed a reduction in recession depth postoperatively. In six months, both groups showed similar MRC double-VISTA with PRF (93.3%) and double-VISTA with SCTG (93.7%). At one year, mean root coverage (MRC) in the double VISTA with platelet-rich fibrin (PRF) group decreased from 93.3% to 75.8%, whereas the subepithelial connective tissue graft (SCTG) group remained stable. SCTG reliably increases tissue biotype (thickness and resilience), which is strongly associated with predictable creeping attachment and long-term margin stability. On the contrary, the faster resorption of PRF, with its notable decrease in cytokine release, could hinder its capacity to maintain prolonged soft-tissue repair. Other factors that determine the stability of recession coverage include anatomical constraints, gingival tissue phenotype, and postoperative patient-related factors (poor oral hygiene maintenance and recurrence of aggressive brushing habits).

Limitations

This case series presents two patients with 12-month follow-up data. Long-term sustainability (>12 months) requires investigation. Patient sample was limited; randomized controlled trials comparing double-VISTA with alternative techniques are necessary. Root-coverage stability depends on postsurgical maintenance and compliance with a modified brushing technique, requiring long-term observation. The overall findings suggest that the double-VISTA approach offers a reliable, minimally invasive, and esthetically favorable option for managing multiple mandibular anterior recession defects while addressing the limitations of the conventional VISTA technique.

## Conclusions

The double-VISTA technique, combined with PRF or SCTG, proved effective for managing multiple Cairo RT1 and RT2 GRs in the mandibular anterior region. This minimally invasive approach offers substantial clinical improvement by promoting root coverage while preserving esthetic outcomes. Within the limitations of this small case series, PRF emerges as a promising alternative to SCTG in the double-VISTA approach, providing comparable therapeutic benefits without the need for harvesting tissue from a secondary donor site, thereby eliminating associated patient morbidity and reducing surgical time and discomfort.

Despite these encouraging results, it is important to acknowledge the need for rigorous long-term prospective studies with larger patient cohorts to comprehensively assess the predictability, sustainability, and overall cost-effectiveness of the double-VISTA technique using PRF compared with traditional root-coverage methods. Such studies should also evaluate patient-reported outcomes and biological healing dynamics to fully establish this approach as a standard of care for treating multiple gingival recessions in challenging anatomical sites.
